# Inhibition of endocytic pathways impacts cytomegalovirus maturation

**DOI:** 10.1038/srep46069

**Published:** 2017-04-13

**Authors:** Madeline A. Archer, Teal M. Brechtel, Leslie E. Davis, Rinkuben C. Parmar, Mohammad H. Hasan, Ritesh Tandon

**Affiliations:** 1 Department of Microbiology and Immunology, University of Mississippi Medical Center, 2500 North State Street, Jackson, MS 39216, USA

## Abstract

Endocytic processes are critical for cellular entry of several viruses; however, the role of endocytosis in cellular trafficking of viruses beyond virus entry is only partially understood. Here, we utilized two laboratory strains (AD169 and Towne) of human cytomegalovirus (HCMV), which are known to use cell membrane fusion rather than endocytosis to enter fibroblasts, in order to study a post-entry role of endocytosis in HCMV life cycle. Upon pharmacological inhibition of dynamin-2 or clathrin terminal domain (TD) ligand association, these strains entered the cells successfully based on the expression of immediate early viral protein. However, both the inhibitors significantly reduced the growth rates and final virus yields of viruses without inhibiting the expression of early to late viral proteins. Clathrin accumulated in the cytoplasmic virus assembly compartment (vAC) of infected cells co-localizing with virus tegument protein pp150 and the formation of vAC was compromised upon endocytic inhibition. Transmission electron micrographs (TEM) of infected cells treated with endocytosis inhibitors showed intact nuclear stages of nucleocapsid assembly but the cytoplasmic virus maturation was greatly compromised. Thus, the data presented here implicate endocytic pathways in HCMV maturation and egress.

Enveloped viruses enter cells using two main pathways, one that involves fusion of the viral envelope with the plasma membrane and a second one that involves endocytosis of the intact virus particles^1^. Entry of HCMV follows direct fusion at the cell surface in fibroblasts but entry into other relevant cell types, such as endothelial cells, follows an endocytic route^2^,^3^. Clathrin is the major constituent of coated vesicles and plays a critical role in the endocytic entry of viruses^1^,^4^,^5^,^6^,^7^,^8^,^9^. Clathrin coated vesicles (CCV) are also important in cell signaling and transport of critical cargo in the cell^10^,^11^,^12^, that can have significant impact on virus replication. The large GTPase dynamin acts as an accessory to clathrin by mediating the scission of the CCV from the parent membrane^13^. CCV selectively sort cargo at the cell membrane, trans-Golgi network (TGN), as well as at the endosomal compartments^14^. The connection of the clathrin scaffold to the membrane is mediated by clathrin adaptors, which can bind directly to both the clathrin lattice and to the lipid and protein components of membranes^15^. Cargo molecules may also be recruited into coated vesicles by direct interactions with clathrin^16^.

Pathogens can hijack cellular adaptors; however, interestingly, there are very few examples to date of viral clathrin adaptor mimics. One such example is the large hepatitis delta antigen (HDAg-L), which functions as a clathrin adaptor to promote hepatitis delta virus assembly. HDAg-L contains a clathrin box motif that can interact with the clathrin heavy chain at the TGN to promote viral morphogenesis^17^,^18^,^19^,^20^. In case of HCMV, major tegument protein pp150 is known to bind to host protein bicaudal D1 (bicD1), which in turn interacts with the dynein motor complex and with Rab-6 GTPase^21^. BicD1 can also interact with clathrin heavy chain (CHC) directly^22^. Depletion of bicD1 leads to reduced viral growth and impaired trafficking of pp150 to the cytoplasmic virus assembly compartment (vAC)^21^. A direct binding of pp150 with CHC has also been demonstrated using an immunoprecipitation - mass spectrometry approach^23^. The mechanisms by which pp150 engages clathrin and the role of this engagement in virus replication are unknown. Clathrin mediated pathways are also known to be involved in exocytosis^24^,^25^,^26^, the primary mechanism by which herpesviruses egress the cells^27^, suggesting a role of clathrin in viral egress as well.

Herpesviruses, including HCMV, can bind to a broad range of cells by engaging cell surface heparan sulfate proteoglycan (HSPG)^2^,^27^. This attachment is believed to initiate a cascade of events involving other cellular receptors and entry mediators that ultimately leads to viral fusion with the host cell membrane either at cell surface or in endosomes. However, mere attachment does not guarantee virus entry or a productive replication cycle. Expression of immediate-early viral proteins (IE) in infected cells can be used as a marker for the successful entry of HCMV but a lack of IE protein expression does not necessarily indicate a defect in virus entry.

Settings where inhibition of endocytosis does not impact virus entry provide an excellent opportunity to investigate the nature and role of endocytic pathways in the late stages of virus replication. Here, we exploited the differences in laboratory and clinical strains of HCMV to reveal a critical aspect of virus maturation where endocytic pathways are important.

## Results

### Inhibition of endocytosis inhibits HCMV replication

HCMV strains Towne and AD169 use a non-endocytic pH-independent fusion mechanism at the cell surface to enter cells^28^. We tested the effect of two different endocytosis inhibitors on replication of these strains in primary human foreskin fibroblasts (HF): (i) Dynasore: The small molecule inhibitor of dynamin-2, which is crucial for endocytic vesicle formation in clathrin- and caveolin-mediated endocytosis^29^ as well as more poorly understood clathrin- and caveolin-independent endocytic pathways^29^,^30^, (ii) Pitstop 2: another small molecule inhibitor that selectively blocks endocytic ligand association with the clathrin terminal domain (TD)^31^. Pitstop 2 also blocks clathrin independent endocytosis however, the mechanism of this block is unknown^32^,^33^.

**Figure 1:**
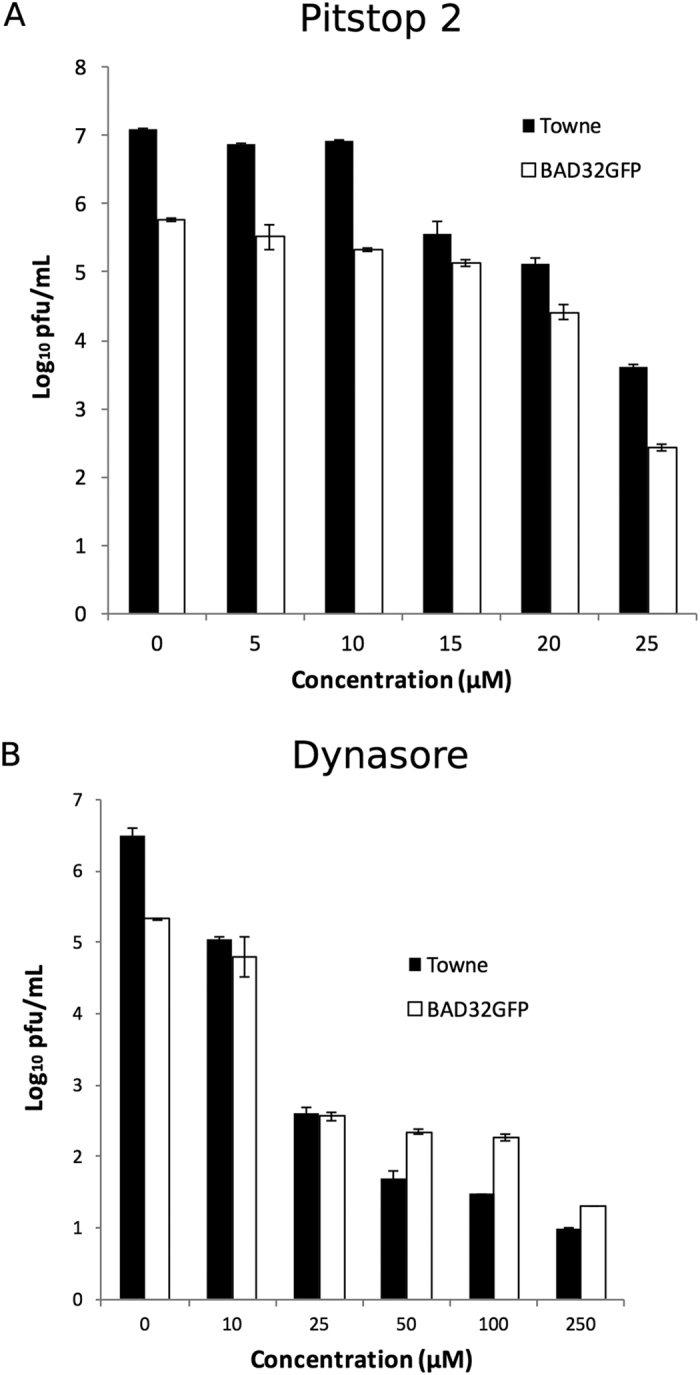
Inhibition of endocytosis impacts the growth of HCMV laboratory strains (Towne and AD169 (BAD32GFP)) in fibroblasts. Confluent HF monolayers were pretreated with dynasore, or pitstop 2 for 1 h, and then infected with Towne or BAD32GFP virus at an MOI of 3.0 in the medium containing the same drug for one hour, washed and thereafter incubated in the presence of the drug. Samples of infected-cells in the cell culture medium were harvested at 5 days post infection and stored at −80 °C before titration. Concentration of pitstop 2 (**A**) in the range of zero to 25 μM or of dynasore (**B**) in the range of zero to 250 μM led to dose dependent inhibition of final virus yields. Triplicate samples were used.

**Figure 2:**
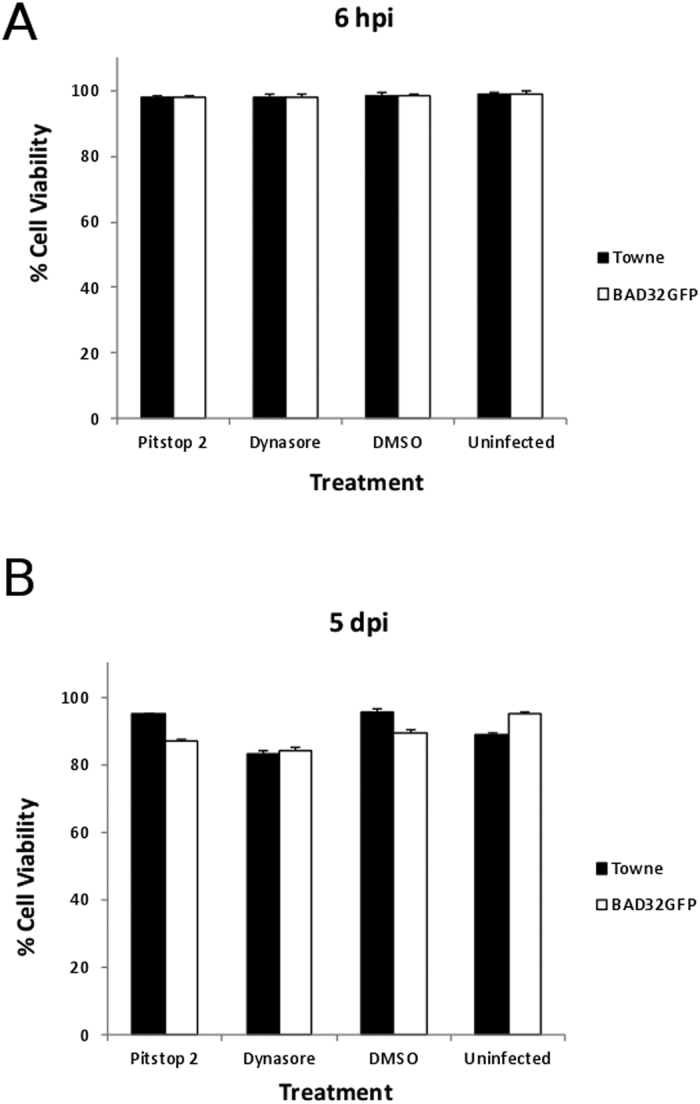
Effect of drug treatments on cell viability. Confluent HF monolayers were pretreated with dynasore (100 μM) or pitstop 2 (25 μM) for 1 h, then infected with Towne or BAD32GFP virus at an MOI of 3.0 in the medium containing the same concentration of the drug for one hour, washed and thereafter incubated for (**A**) 6 hours or (**B**) 5 days in the presence of the same concentration of the drug. DMSO-treated infected cells and uninfected untreated cells served as controls in this experiment. Cell viability was determined using trypan blue exclusion assay as described in materials and methods. Triplicate samples were used.

An IC_50_ value of 12 μM of pitstop 2 has been reported for the inhibition of amphiphysin-1 and clathrin TD association^31^. An increasing concentration of pitstop 2 in the range of zero to 25 μM led to dose-dependent decrease in final virus yields for both Towne and AD169 (BAD32GFP) viruses (Fig. 1A). For dynasore, an IC_50_ value of 15 μM has been reported for the inhibition of dynamin functions^29^. Concentrations of dynasore in the range of zero to 250 μM resulted in a dose dependent inhibition of final virus yields (Fig. 1B). The reduction in virus yield was not due to an impact of these drugs on cell viability (Fig. 2). When HFs were incubated with the inhibitory concentration of these drugs (100 μM dynasore or 25 μM pitstop 2,) for a period of 6 hours post infection (Fig. 2A) or up to 5 days post infection (Fig. 2B), they did not show any significant cell death compared to DMSO control or uninfected cells. These results demonstrate that two different inhibitors that use different mechanisms to inhibit endocytosis inhibit the growth of laboratory strains of HCMV.

### Cellular entry of HCMV laboratory strains does not involve endocytic mechanisms

**Figure 3:**
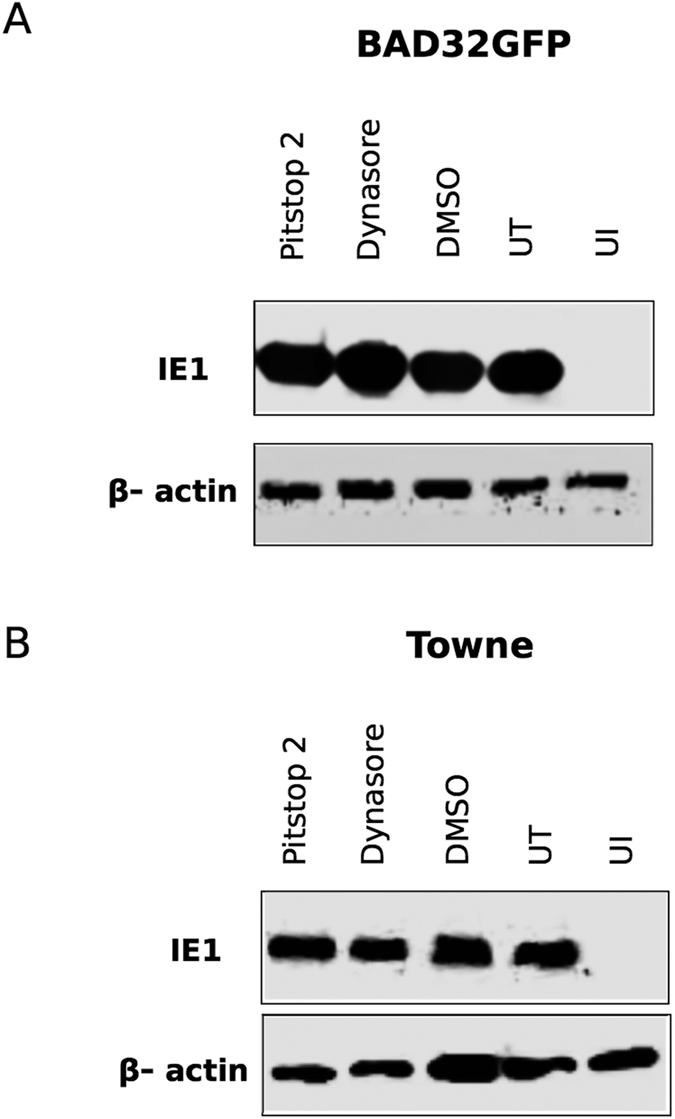
Entry of HCMV laboratory strains AD169 (BAD32) (**A**) and Towne (**B**) in HF upon treatment with pitstop 2, or dynasore as determined by expression of viral immediate early 1 (IE1) protein in infected cells. Confluent HF monolayers were pretreated with dynasore (100 μM) or pitstop 2 (25 μM) for 1 h, then infected with BAD32GFP or Towne virus at an MOI of 3.0 in the medium containing the same concentration of the drug for one hour, washed and thereafter incubated for 6 hours in the presence of the same concentration of drug before harvesting for immunoblots. Triplicate samples were used. DMSO-treated infected cells, untreated infected cells (UT) and untreated uninfected cells (UI) served as controls in this experiment. β-actin was used as a loading control. Immunoblots were digitally cropped to conserve the space.

It has been reported that laboratory strains of HCMV do not require endocytosis for entry into cells^28^. Expression of viral immediate early (IE) protein was analyzed in the lysates of infected cells at 6 hours post infection (Fig. 3A,B). Both BAD32GFP (AD169) and Towne strains of HCMV entered HF cells upon treatment with pitstop 2 or dynasore based on equivalent amount of IE1 protein expression in mock-treated and drug-treated cells.

### Viral protein expression is not eliminated upon endocytic inhibition in fibroblasts

**Figure 4:**
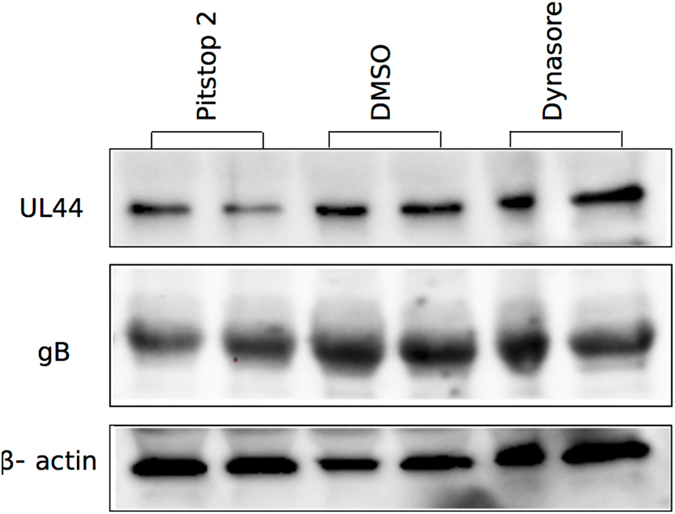
Pitstop 2 or dynasore treatment does not eliminate the expression of viral early to late proteins in infected cells. Confluent HF monolayers were pretreated with dynasore (100 μM), pitstop 2 (25 μM), or mock (DMSO) for 1 h, then infected with BAD32GFP virus at an MOI of 3.0 in the medium containing the same concentration of the drug for one hour, washed and thereafter incubated for 72 hours in the presence of the same concentration of drug before harvesting for immunoblots using antibodies against early (pUL44) and late (gB) viral antigens. Duplicate samples were used for each treatment. DMSO-treated infected cells served as mock-control in this experiment and β-actin was used as a loading control. Immunoblots were digitally cropped to conserve the space.

To determine the impact of inhibition of endocytosis on post entry phases of HCMV life cycle, we probed the expression of early (pUL44) and late (gB) viral proteins. Both of these proteins were expressed when HFs were infected in the presence of pitstop 2 or dynasore at inhibitory concentrations (Fig. 4). Although some differences were observed in the expression levels of these proteins, any significant impact of these drugs on early stages of virus infection can be ruled out based on this data.

### Clathrin concentrates in vAC and co-localizes with the vAC marker pp150

**Figure 5:**
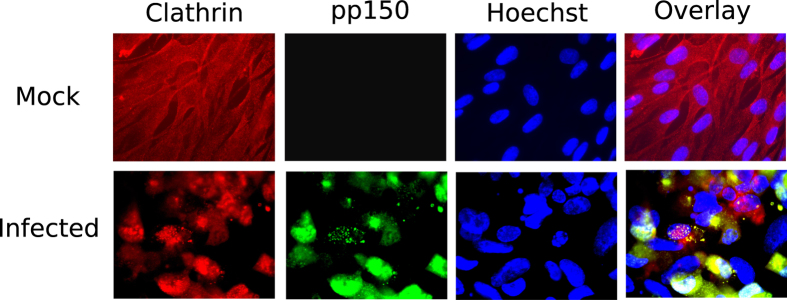
Co-localization of clathrin and pp150 during HCMV infection. HF were mock-infected or infected with BAD32GFP virus at an MOI of 3.0 and fixed for IFA at 4 days post infection. Shown are the groups of four panels (mock or infected), obtained from the same field that includes single-color images of the cellular antigen (red), viral antigen pp150 (green), DNA (in nuclei) detected by Hoechst 33258 (blue, middle panels), or a composite (overlay, right). Clathrin heavy chain antibody was used for clathrin detection and the GFP fluorescence from BAD32GFP virus marked pp150. Hoechst staining marked the nuclei.

**Figure 6:**
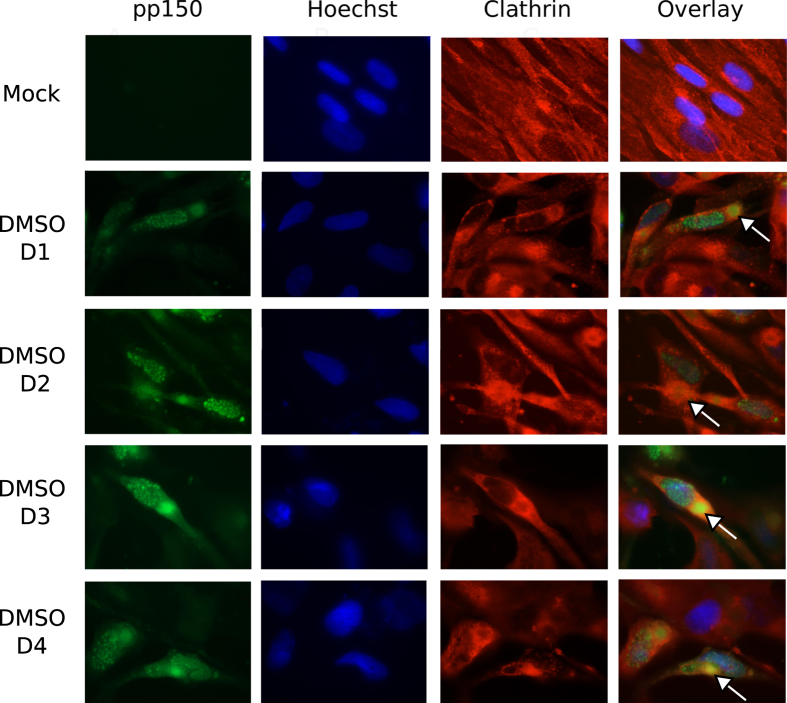
Co-localization of clathrin and pp150 during HCMV infection when cells were mock treated at infection or at consecutive days post infection. HF were mock-infected or infected with BAD32GFP virus at an MOI of 3.0 and fixed for IFA at 5 days post infection. DMSO was added at day 1 (at the time of infection) or at days 2, 3, 4 of infection. Shown are the groups of four panels (mock or infected), obtained from the same field that includes single-color images of the cellular antigen (red), viral antigen pp150 (green), DNA (in nuclei) detected by Hoechst 33258 (blue), or a composite (overlay, right). Clathrin heavy chain antibody was used for clathrin detection and the GFP fluorescence from BAD32GFP virus marked pp150. vAC are marked with an arrow in the overlay image.

**Figure 7:**
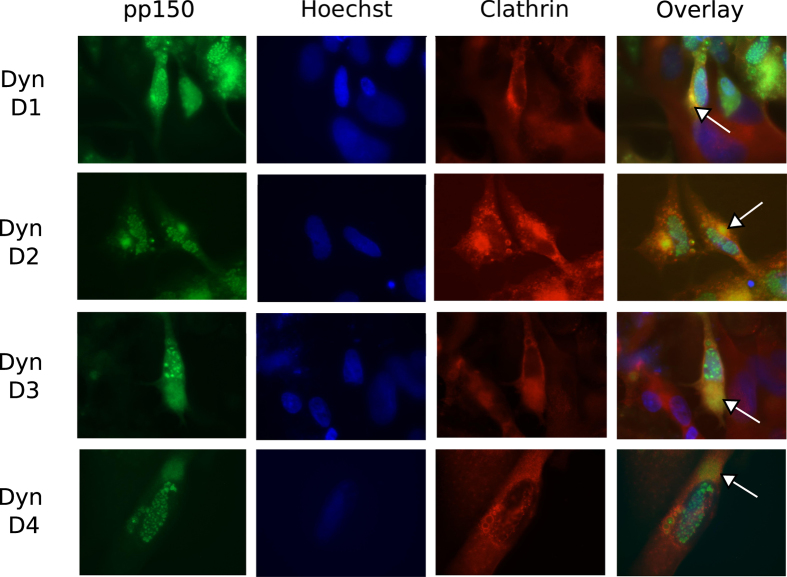
Co-localization of clathrin and pp150 during HCMV infection when cells were dynasore treated at infection or at consecutive days post infection. HF were mock-infected or infected with BAD32GFP virus at an MOI of 3.0 and fixed for IFA at 5 days post infection. Dynasore was added at day 1 (at the time of infection) or at days 2, 3, 4 of infection. Shown are the groups of four panels (mock or infected), obtained from the same field that includes single-color images of the cellular antigen (red), viral antigen pp150 (green), DNA (in nuclei) detected by Hoechst 33258 (blue), or a composite (overlay, right). Clathrin heavy chain antibody was used for clathrin detection and the GFP fluorescence from BAD32GFP virus marked pp150. vAC are marked with an arrow in the overlay image.

**Figure 8:**
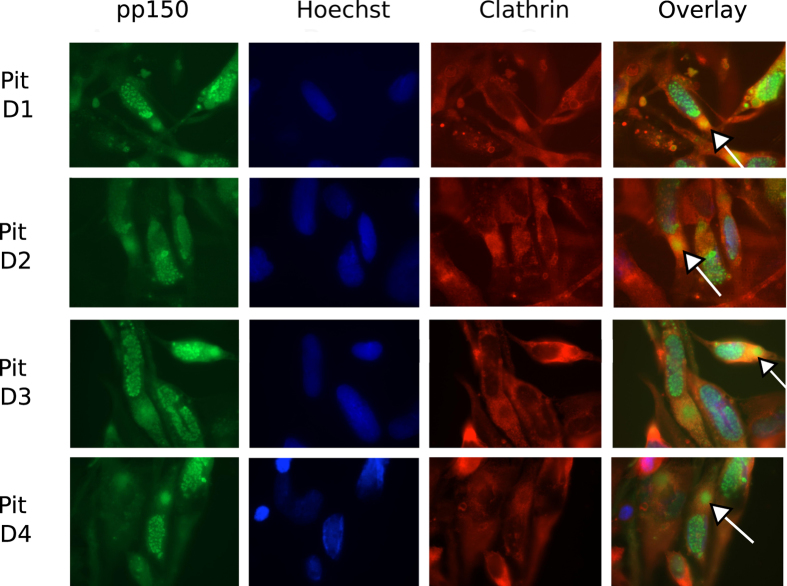
Co-localization of clathrin and pp150 during HCMV infection when cells were pitstop 2 treated at infection or at consecutive days post infection. HF were mock-infected or infected with BAD32GFP virus at an MOI of 3.0 and fixed for IFA at 5 days post infection. Pitstop 2 was added at day 1 (at the time of infection) or at days 2, 3, 4 of infection. Shown are the groups of four panels (mock or infected), obtained from the same field that includes single-color images of the cellular antigen (red), viral antigen pp150 (green), DNA (in nuclei) detected by Hoechst 33258 (blue), or a composite (overlay, right). Clathrin heavy chain antibody was used for clathrin detection and the GFP fluorescence from BAD32GFP virus marked pp150. vAC are marked with an arrow in the overlay image.

A direct interaction between clathrin heavy chain and pp150 has been reported earlier^23^. We followed the localization pattern of clathrin during HCMV infection in immunoflurescent assays using a monoclonal antibody against clathrin heavy chain (CHC, BF-06). Clathrin localized in punctate cytoplasmic structures in mock-infected cells but concentrated in vAC of infected cells (Fig. 5), where it co-localized with the tegument protein pp150 (Fig. 5 overlay). pp150-GFP is expressed from the virus genome here; thus, labeling of vAC by pp150-GFP is not influenced by the assay conditions. It is possible that pp150 or other viral proteins directly or indirectly (through binding with clathrin adaptors) engage clathrin for the purpose of virus trafficking in the cell. We followed the clathrin and pp150 localization over the time-course of drug addition in infected cells. Clathrin distributes evenly in the cytoplasm of mock-infected HF and can be visualized as punctate structures, some which concentrate at the perinuclear region (Fig. 6, top panel). Upon infection, in DMSO-treated cells, clathrin localizes in vAC (arrows) along with pp150 irrespective of the time of DMSO treatment (day 1 to day 4 of infection). Treatment with dynasore impacts the formation of vAC when dyansore is added at early times post infection (day 1 of infection) but not when dynasore is added at late times post infection (day 2 to day 4 of infection) (Fig. 7) indicating that the vAC starts to assemble early during infection. Nevertheless, the co-localization of pp150 and clathrin to vAC is not disrupted upon dynasore treatment at any time post infection. Pitstop 2 treated cells seem to form a rather compact vAC, very much like mock-treated cells, and this vAC or pp150- clathrin co-localization is not affected by the time of addition of Pitstop 2 (Fig. 8). Addition of dynasore at early times post infection (day 1 and day 2) but not at late times post infection (day 3 and day 4) led to a decrease in the number of viral plaques (Fig. S1). Also, gB localization seemed impaired when dynasore was added at the time of infection but not with pitstop 2 treatment in the same manner (Fig. S2).

### Late stages of cytoplasmic virus maturation are impaired upon dynamin and clathrin inhibition

**Figure 9:**
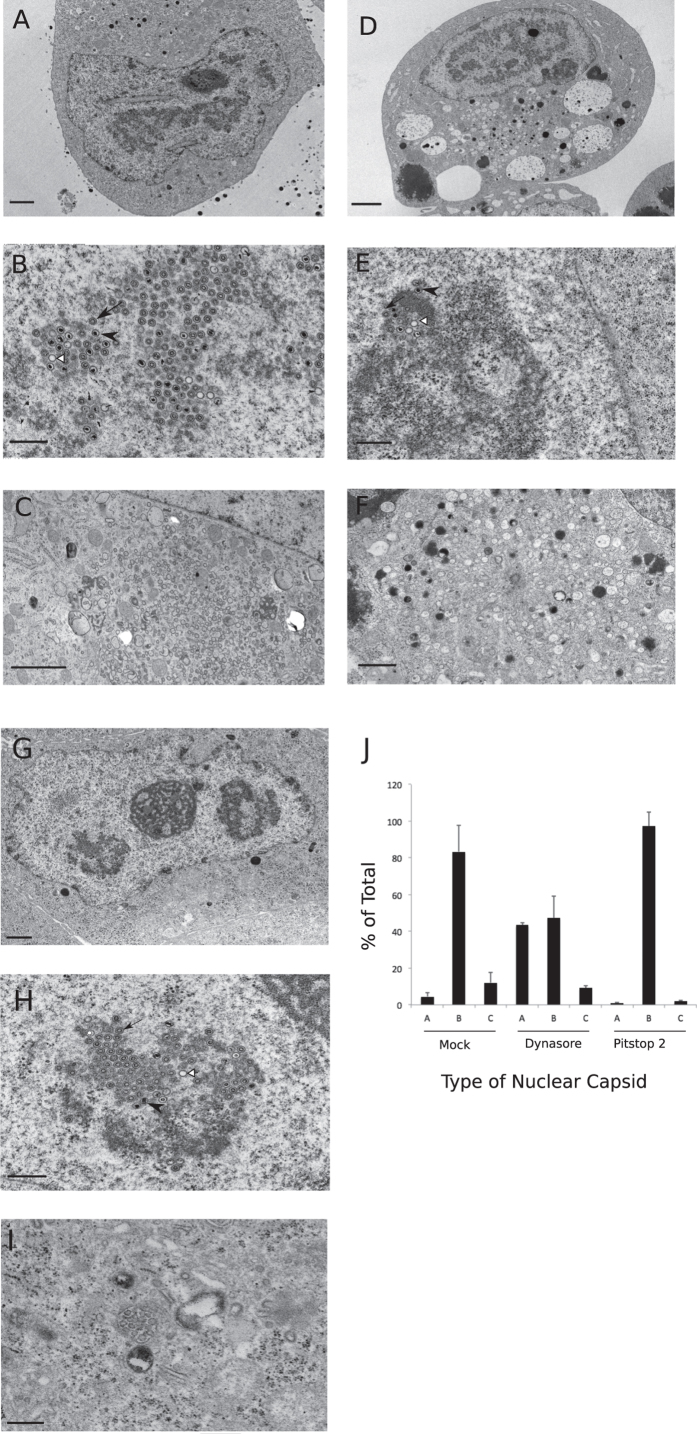
Transmission electron micrographs of HF cells that were either mock-treated (**A**,**B** and **C**) or treated with dynasore (100 μM) (**D**,**E** and **F**) or pitstop 2 (25 μM) (**G**,**H** and **I**) and then infected with HCMV (AD169) at an MOI of 3.0 and incubated in the presence of indicated drug for 4 days before processing for TEM. Representative whole cell (**A**,**D**,**G**), nuclear (**B**,**E**,**H**) or cytoplasmic (**C**,**F**,**I**) sections of the infected cell are shown in the micrographs. (**J**) Quantification of nuclear capsid types from the above treatments. A total of 400, 318 or 674 capsids were counted for mock, dynasore and pitstop 2 treatments, respectively. Scale: 2 μm (**A**,**C**,**D**), 1.0 μm (**F**,**G**), 0.5 μm (**B**,**E**,**H**,**I**). A- (white arrowheads), B- (black arrows), and C- capsids (black arrowheads).

Transmission electron microscopy of HCMV (AD169) infected HF that were mock-treated or treated with dynasore or pitstop 2 revealed several differences. Mock-treated cells showed a typical kidney-shaped nucleus observed in HCMV infected cells (Fig. 9A) and all three types of nuclear capsids, namely A, B- and C- capsids were present (Fig. 9B)^34^. In the cytoplasm, several mature and immature virus particles could be visualized in an area corresponding to vAC (Fig. 9C). For the dynasore-treated cells, the cells took the typical morphology of HCMV infected cell; however, several large, seemingly empty or debris-containing cytoplasmic vesicles could be visualized (Fig. 9D). Dynasore-treated cells contained all three types of nuclear capsids (Fig. 9E); however, the cytoplasm contained few, if any mature virus particles (Fig. 9F). For pitstop 2, the cell took the typical morphology of an infected cell (Fig. 9G) and all three nuclear capsids were detected (Fig. 9H) but the cytoplasm lacked any discernable mature virus particles (Fig. 9I).

Quantification of nuclear capsids showed differences in the proportions of A-, B- and C- capsids among these treatments with mock- and pitstop 2 treated cells having an overwhelming majority of B-capsids and dynasore treated cells showing significant increase in the proportion of A-capsids (Fig. 9J). Most importantly, the genomic DNA containing C-capsids were present in all treatments although the numbers were reduced in pitstop 2 treated cells (Fig. 9J). Presence of very few particles and that too with irregular morphology prevented the absolute quantification and comparison of particles in the cytoplasm of treated-infected cells.

## Discussion

Virus trafficking in host cells depends upon a variety of host factors ranging from cytoskeletal network to endocytic machinery. While the role of endocytosis in virus entry has been studied extensively, little knowledge exists on its role in virus trafficking post entry. We used a system where virus entry does not depend upon endocytosis to investigate the functions of host endocytic machinery in virus trafficking and maturation. The results suggest that these host components are required at a late stage of HCMV maturation after synthesis of late viral proteins. Earlier, we reported the involvement of endosomal sorting complex required for transport (ESCRT) machinery in HCMV maturation^35^ and recent studies have strongly indicated that endocytic and exocytic pathways are exploited in the process of virus maturation, envelopment and egress^26^,^36^,^37^,^38^,^39^,^40^,^41^,^42^. This manuscript provides evidence to directly link the endocytic pathways to HCMV maturation. Use of two different endocytic inhibitors (dynasore and pitstop 2) led to significant decrease in HCMV titers without impacting early to late virus gene expression. We probed the localization of clathrin during infection and not surprisingly it was found to concentrate in vAC, the site of endosome accumulation as well as virus maturation and envelopment 35,43,44. Clathrin co-localized with pp150, a viral tegument protein known to be critical for late phase of virus maturation^44^,^45^. When dynasore was added at an early time post infection, the vAC was less developed and the virus growth was significantly reduced. A direct interaction between pp150 and clathrin heavy chain has been reported^23^, indicating that HCMV may use pp150 to directly engage clathrin.

The TEM experiments confirmed that the nuclear stages of capsid assembly and maturation were intact and the genomic DNA containing C-capsids were present in all treatments although the numbers were significantly reduced for pitstop 2 treated cells. Clathrin is important in cell signaling and other activities^10^,^11^,^12^ that can have significant impact on HCMV replication prior to the stage of nucleocapsid assembly as well. A- and B-capsids are both considered intermediate or abortive stages in the process of nucleocapsid assembly^34^ and it would be interesting to explore why dynasore treatment led to an increase in the relative proportion of A-capsids without a significant decrease in the proportion of C-capsids compared to the mock-treated cells.

Other known scenario where endocytic pathways are important for virus maturation is virus glycoprotein trafficking. The de-envelopment – re-envelopment model of herpesvirus envelope acquisition requires that mature glycoproteins traffic to endosomes and/or TGN to be incorporated into virus envelope^46^. This involves, at least in the case of Varicella Zoster virus (VZV), endocytosis of mature virus glycoproteins from the plasma membrane via a clathrin and dynamin dependent process^46^,^47^,^48^,^49^. Also, due to dynamic interconnection between endocytic and exocytic pathways, viral proteins recovered from the plasma membrane could be used by trans-Golgi or endosomal cisternae to form new viral envelopes. Adherence of enveloped virions to unrecycled viral proteins on the cell surface in the absence of clathrin-mediated endocytosis may also contribute to decreased virus release^26^.

The data presented here provides first glimpses into the role of endocytic pathways in HCMV maturation and egress. Although pox viruses are known to use cytoskeleton for egress^50^, the role of cytoskeletal proteins in cytomegalovirus maturation has not been addressed yet. In fact, cytoskeletal transcripts are downmodulated during late phases of CMV infection^51^. The pharmacological inhibition studies like the present study may suffer from possible off-target effects of the drugs; however, it is important to note that the specific activities of dynasore and pitstop 2 are fairly well established^29^,^31^,^52^ and we have used these two different endocytic inhibitors with different mechanisms of action in parallel to come to the same conclusion. Pharmacologic inhibition of the endocytosis is also a promising strategy to develop antivirals^53^. Future studies will look into specific inhibition of biological pathways by siRNA, dominant negative mutants or knockout cell lines to confirm the findings presented in this study. We hope these studies collectively will open up the possibilities of designing an antiviral strategy by blocking the engagement of endocytic pathways by HCMV in addition to advancing our knowledge on host pathogen interaction and specific steps in herpesvirus maturation.

## Materials and Methods

### Cells

Primary human foreskin-derived fibroblasts (HF) were cultured in Dulbecco’s modified Eagle’s medium (DMEM, Invitrogen Corporation, Carlsbad, CA) containing 4.5 g/ml glucose, 10% fetal bovine serum (#S1245OH; Atlanta Biologicals, Lawrenceville, GA), 1 mM sodium pyruvate, 2 mM L-glutamine, and 100 U/ml penicillin-streptomycin (Cellgro, Manassas, VA) at 37 °C with 5% CO_2_. HFs between passages 5 and 15 were used for transfections and infections. The cell culture medium was changed every other day in infections with new drugs added. The viability of cells was determined by trypan blue exclusion assay using established protocols^54^ and recorded on TC10 automated cell counter (BioRad, Hercules, CA).

### Antibodies, immunofluorescence assays and immunoblots

Anti clathrin heavy chain antibody (BF-06, Thermo Scientific Pierce, Rockford, IL) was used as the primary antibody in immunofluorescence assays. Mouse monoclonal antibodies to gB (2F12), pUL44 (ICP36), and IE1 (CH160) were purchased from Virusys corporation, Sykesville, MD, USA. Fluorescent label tagged secondary antibody DYLIGHT 594 was purchased from Thermo Scientific Pierce and used in immunofluorescent assays (IFA) described below. Hoechst 33258 (Thermo Scientific Pierce) staining identified the nuclei in IFA. Anti β-actin antibody (AC-74, Sigma-Aldrich, St Louis, Mo, USA) was used as a control for sample loading in immunoblots. Peroxidase-labeled horse anti-mouse IgG (Vector Laboratories, Burlingame, CA) was used as the secondary antibody for IBs. Blots were detected using ECL Western blotting detection reagents (GE Healthcare, Buckinghamshire, United Kingdom).

### Drug inhibition experiments

Confluent HF monolayers were pretreated with dynasore (#324410, Merck Millipore, Billerica, MA) or pitstop 2 (# ab120687, Abcam, Cambridge, MA) for 1 h, then infected with Towne or BAD32GFP^23^ virus at an MOI of 3.0 in medium containing drug for one hour, washed and thereafter incubated for up to 5 days in the presence of drug. Triplicate samples were used for all drug block studies. Samples of infected-cells along with the medium were harvested at designated time points and stored at −80 °C before titration by fluorescent focus assays on HF.

### Microscopy

Samples were prepared using established protocols for IFA and confocal fluorescence microscopy. Briefly, cells (HF) were grown on coverslip-inserts in 24 well tissue culture dishes and infected with an MOI of 3.0 at confluency. At the end point of experiment, cells were fixed in 3.7% formaldehyde for 10 min and were incubated in 50 mM NH_4_Cl in 1X PBS for 10 min to reduce autofluorescence. This was followed by washing in 1X PBS, incubation in 0.5% Triton X-100 for 20 min to permeabilize the cells and finally washing and incubation with primary and secondary antibodies at 1:1000 dilution in 0.1% bovine serum albumin in 1X PBS. Coverslips were retrieved from the wells and were mounted on glass slides with a drop of mounting medium (Gel/Mount, Biomeda, Foster city, CA) and dried overnight before imaging. Images were acquired on Zeiss Axio Imager A1 epifluorescent microscope using 40X or 100X objective. Samples for TEM were prepared by fixing the cells (HF) at endpoint in 2.5% glutaraldehyde in 0.1 M cacodylate buffer (pH 7.2) for 2 h at room temperature. Cells were then washed with the same buffer and postfixed with buffered 1.0% osmium tetroxide at room temperature for 1 h. Following several washes with 0.1 M cacodylate buffer, cells were dehydrated with ethanol, infiltrated, and embedded in Eponate 12 resin (Ted Pella Inc., Redding, CA). Cell culture plates were cracked with a hammer to release the resin after it had solidified, and ultrathin sections (60 to 70 nm) of monolayer cells were cut and counterstained using uranyl acetate and lead citrate. Examination of ultrathin sections was carried out on a Hitachi H-7500 TEM operated at 75 kV, and images were captured using a Gatan BioScan (Pleasanton, CA) charge-coupled device camera. The images were acquired and analyzed with the Digital Micrograph (Pleasanton, CA) software.

## Additional Information

**How to cite this article:** Archer, M. A. *et al*. Inhibition of endocytic pathways impacts cytomegalovirus maturation. *Sci. Rep.* **7**, 46069; doi: 10.1038/srep46069 (2017).

**Publisher's note:** Springer Nature remains neutral with regard to jurisdictional claims in published maps and institutional affiliations.

## Supplementary Material

Supplementary Information
