# Supplementary material for: Inhibition of endocytic pathways impacts cytomegalovirus maturation
Source: Sci Rep. 2017 Apr 13;7:46069. doi: 10.1038/srep46069 (PMC5390266; doi:10.1038/srep46069)

Supplementary Information

Inhibition of endocytic pathways impacts cytomegalovirus maturation

Madeline A. Archer, Teal M. Brechtel, Leslie E. Davis' Rinkuben C. Parmar, Mohammad H. Hasan, and Ritesh Tandon

Fig. S1. Inhibition of endocytosis at early stages of infection impacts the growth of HCMV in fibroblasts. Confluent HF monolayers were pretreated with 100 µM dynasore or DMSO (day 1) and then infected with BAD32GFP virus at an MOI of 3.0 in the medium containing the same drug for one hour, washed and thereafter incubated for 5 days in the presence of the drug. Alternatively, HF were infected with BAD32 virus at an MOI of 3.0 and dynasore was added at days 2, 3 or 4 of infection and maintained until harvest. Triplicate samples were used. Samples of infected-cells in the cell culture medium were harvested at 5 days post infection and stored at -80°C before titration on fresh fibroblasts.

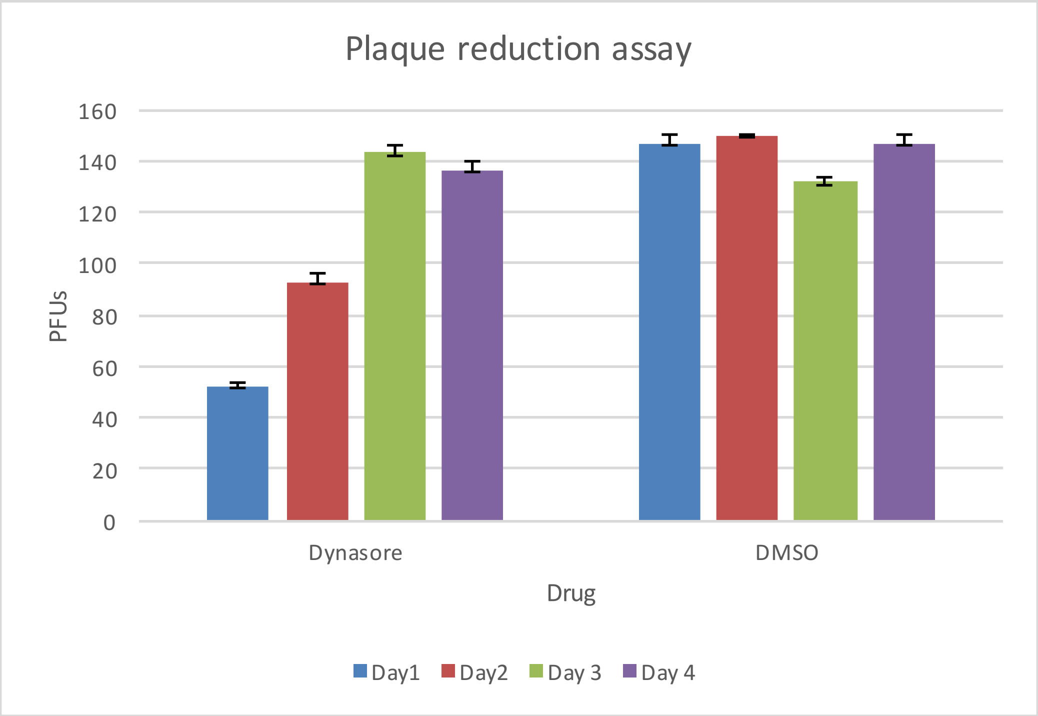

Fig S2. Expression and localization of viral glycoprotein gB and tegument protein pp150 during HCMV infection marking the vAC. HF were infected with BAD32GFP virus at an MOI of 3.0 and fixed for IFA at 5 days post infection. DMSO (mock), dynasore (Dyn) or pitstop 2 (Pit) were added at day 1 (at the time of infection) and maintained until fixation. Shown are the groups of four panels, obtained from the same field that includes single-color images of gB (red), pp150 (green), DNA (in nuclei) detected by Hoechst 33258 (blue), or a composite (overlay, right). gB localizing to vAC is marked with an arrow.

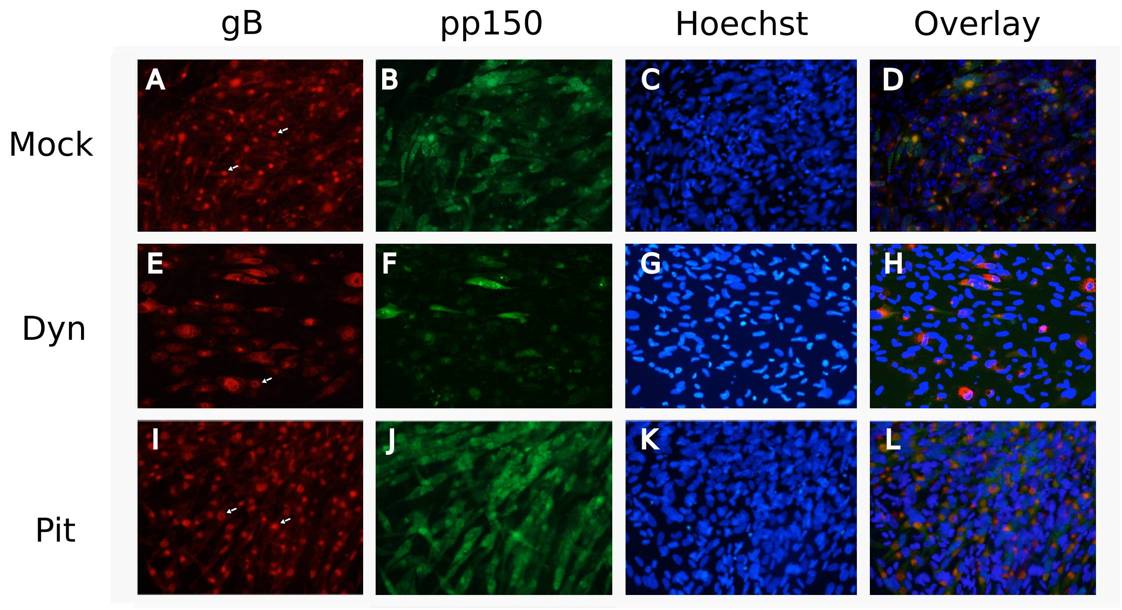

Supplement: Supplementary Information [file srep46069-s1.pdf]
